# Potential of biosynthesized zinc oxide nanoparticles to control *Fusarium* wilt disease in eggplant (*Solanum melongena*) and promote plant growth

**DOI:** 10.1007/s10534-022-00391-8

**Published:** 2022-03-31

**Authors:** Amer M. Abdelaziz, Salem S. Salem, Ahmed M. A. Khalil, Deiaa A. El-Wakil, Hossam M. Fouda, Amr H. Hashem

**Affiliations:** 1 Department of Botany and Microbiology, Faculty of Science, Al-Azhar University, Nasr City, 11884 Cairo Egypt; 2 Biology Department, College of Science, Taibah University, Yanbu, 41911 Kingdom of Saudi Arabia; 3 Department of Biology, Faculty of Science, Jazan University, Jizan, 82817 Kingdom of Saudi Arabia; 4 Plant Pathology Research Institute, Agricultural Research Center, Giza, 12619 Egypt

**Keywords:** *Fusarium oxysporum*, Eggplant, *Solanum melongena*, Wilt and ZnO-NPs

## Abstract

In this study, a novel, non-toxic, eco-friendly zinc oxide nanoparticles (ZnO-NPs) was used instead of the synthetic fungicides widely used to control the destructive phytopathogenic fungus *Fusarium oxysporum*, the causative agent of wilt disease in *Solanum melongena* L. Herein, the biosynthesized ZnO-NPs was carried out by *Penicillium expansum* ATCC 7861. In vitro, mycosynthesized ZnO-NPs exhibited antifungal activity against *Fusarium oxysporum*. In vivo, ZnO-NPs suppressed *Fusarium* wilt disease in cultivated *Solanum melongena* L. by decreasing the disease severity with 75% of plant protection. Moreover, ZnO-NPs stimulated the recovery of eggplant as an indicated by improving of morphological and metabolic indicators including plant height(152.5%), root length(106.6%), plant fresh biomass (146%), chlorophyll a (102.8%), chlorophyll b (67.86%), total soluble carbohydrates (48.5%), total soluble protein (81.8%), phenol (10.5%), antioxidant activity and isozymes compared with infected control**.** Therefore, this study suggests using mycosynthesized ZnO-NPs as an alternative to synthetic fungicides not only to eradicate the *Fusarium* wilt disease in cultivated eggplant (*Solanum melongena*) but also to promote the growth parameters and metabolic aspects.

## Introduction

Eggplant (*Solanum melongena *L.) (*S. melongena*) is a vital vegetable crop of the temperate and tropical region of the world. Egypt is the third country worldwide in eggplant production after China and India (Sulaiman et al. 2020). Eggplant has antioxidant activities that promote heart health (Rodriguez-Jimenez et al. 2018). Additionally, it contains a great source of fiber with low concentrations of water-soluble carbohydrates that regulate blood sugar and control glucose level, along with the phenolics in eggplant that inhibit enzymes involved in type 2 diabetes (Kwon et al. 2008). Eggplant is a reservoir of calcium, magnesium, iron, polyphenolic compounds, phytonutrients as fatty acids, chlorogenic acid, amino acids, vitamins that help improve human health (Sharma and Kaushik 2021). Moreover, eggplant act as a model plant for studying plant physiology and genetics (Magioli and Mansur 2005). Most cultivated varieties of Eggplant are susceptible to a large number of pathogens, especially fungi that cause huge serious losses in crop production (Magioli and Mansur 2005). *Fusarium oxysporum* has the ability to survive in the soil for a long period of about two decades, and thus it is classified as the most destructive soil-borne fungus that affects most crops, especially eggplant (Gordon and Martyn 1997). *Fusarium* is the most common soil-borne pathogen that invades many vegetables such as tomatoes, potatoes, peppers, and eggplant. The pathogen *Fusarium oxysporum* attacks plant through the roots and spreads to the stems and leaves, which limits the flow of water, causing the leaves to wilt and turn yellow (Najar et al. 2011). In eggplants, *Fusarium* wilt appears as slight vein clearing on the outer portion of the young leaves often on one side of the plant or on shoot and successive leaves yellow wilt and die, often before the plant reaches maturity. As the disease progresses, plant stunted, and little or no fruit develops. The browning of the vascular system is characteristic of diagnostic of *Fusarium* wilt (Miller et al. 2008). Nanotechnology has contributed to mitigated challenges in plant disease management by reducing chemical inputs, promoting plant growth and improving biomass production to contribute to meet growing global needs (Elmer and White 2018; Eid et al. 2021). Zinc is widely used in the control of plant diseases, especially those caused by fungi, while zinc oxide nanoparticles (ZnO-NPs) are more effective in suppressing the growth of plant fungal pathogens (Khan et al. 2019). Previous studies showed the ability of ZnO-NPs to control fungal plant diseases such as *Fusarium oxysporum* through their ability to directly inhibit fungal growth by distorting the growing mycelia and also by eliminating mycotoxins such as fusaric acid (Yehia and Ahmed 2013). The efficacy of ZnO-NPs as antifungal and anti-mycotoxin against *Fusarium sp* may be due to upraised the lipid peroxidation, reactive oxygen species (ROS) levels and changing in the ergosterol content that altered the membrane integrity and morphology of macro conidia (Ramachandrappa et al. 2019). Nanotechnology has the potential to develop all areas of science to achieve their highest levels because of their clear and distinct effects that provide the scientific community with many developments that have an applied impact in the medical, agricultural, and many other fields (Hassan et al. 2019; Eid et al. 2020; Fouda et al. 2020; Salem et al. 2020; Hashem et al. 2021a, b; Hashem and Salem 2022; Salem et al. 2022; Sharaf et al. 2022). Fungi are one of the most important group of microbes, as they are used in many applications such as bio-processing, removal of bio-inks & dyes, enzyme production, lipid production, food products, and nanotechnology (Fouda et al. 2019; Hashem et al. 2019; Salem et al. 2019; Hasanin et al. 2020; Hashem et al. 2020a, b; Hashem et al. 2020a, b; Shaheen et al. 2021a, b). Biological synthesis of metal nanoparticles is stable, biologically safe and ecofriendly (Salem and Fouda 2021; Shaheen et al. 2021a, b; Salem 2022). ZnO-NPs have the potential to improve the production and growth of food crops through their use as fertilizer in soil as well as foliar fertilizer (Milani et al. 2012). ZnO-NPs applied in agricultural field as Nano bio fertilizer to enhance the quality and quantity of the yield as well as a protective agent against plant pathogens (Singh et al. 2018). Recently, ZnO-NPs have been frequently used not only for controlling plant pathogens but also for their positive role in soil fertility and reducing toxicity (Dimkpa et al. 2012). Thunugunta et al. (2018) reported the potency of ZnO-NPs to boost growth of eggplant under greenhouse conditions through enhancement of seed germination, photosynthetic pigments, carbohydrates, protein, and increasing antioxidant enzymes activity (Singh et al. 2013). Therefore, this study aimed to myco-synthesis of ZnO- NPs by *Penicillium expansum* ATCC 7861 and zinc acetate as the precursor to Zn. ZnO-NPs were investigated and characterized by TEM, DLS, SEM–EDX, Ft-IR, and XRD. An attempt was made to investigate the antifungal activity of ZnO-NPs against the soil-born pathogen *F. oxysporum* in vitro. Additionally, ZnO-NPs were investigated for use in controlling *Fusarium* wilt disease of eggplant and promoting plant growth in vivo.

## Materials and methods

### Extracellular biosynthesis of ZnO-NPs

#### Cell filtrate preparation

Spore suspension of *Penicillium expansum* was inoculated in Czapek Dox (CD) as broth media used for fermentation process at 30 ± 2 °C for 96 h in an orbital shaker (120 rpm). The biomass was harvested by passing through three layers of lawn cloth and then washed with sterilized distilled water to remove any medium components and about 10 g was suspended 100 ml distilled water. The mixture was agitated for 48 h at 30 ± 2 °C. Finally, the biomass filtrate was obtained by passing it through Whatman filter paper no.1, and then centrifuged at 1000 rpm for 5 min. to sediment any cell debris. This supernatant was used to produce ZnO-NPs.

#### Biosynthesis of ZnO-NPs by biomass filtrate

The previously fungal extract (biomass filtrate) was used for biosynthesis of ZnO-NPs as the following: 2 mM zinc acetate was mixed with 100 ml of biomass filtrate in a 250 ml flask and incubated at 28 ± 2 °C for 24 h, agitated at 150 rpm **(**Fouda et al. 2018**)**. Negative controls (cell filtrate or zinc acetate solution) were also run along with the experiment.

### Characterization of ZnO-NPs

Characterization of ZnO-NP was done by TEM (JEOL 1010 Japan) to know the size and shape of nanoparticles. The sample was prepared by drop-coating the ZnO-NPs solution onto the carbon-coated copper grid and was loaded onto a specimen holder. TEM micrographs were taken and then sizes and shape of ZnO-NPs was confirmed. The particle sizes distribution of ZnO-NPs were evaluated using DLS) measurement conducted with a Malvern Zetazier Instrument. Measurements were taken in the range between 0.1 and 1000 nm. Data obtained were analyzed using Zetasizer software. The elemental composition of mycosynthesized ZnO-NPs was explored using SEM (JEOL, JSM-6360LA, Japan), which was connected with an energy dispersive spectroscopic (EDX) instrument. A known weight of ZnO-NPs one mg was taken in a mortar and ground with dry 2.5 mg of KBr. The powder so obtained was filled in a 2 mm internal diameter micro-cup and loaded onto Ft-IR set at 26 °C ± 1 °C. The sample was scanned using infra-red in the range of 400–4000 cm^1^ utilizing Fourier Transform-Infrared Spectrometer. X-Ray diffraction-patterns were obtained with the XRD 6000 series, including: stress analysis, residual-austenite quantitation, crystallite size/lattice, crystallite calculation, and materials analysis by overlaid X-ray diffraction-patterns Shimadzu-apparatus using filter of nickel and target of Cu-Ka, Shimadzu-Scientific Instruments (SSI), Kyoto Japan. The average crystallite size of ZnO-NPs can also be measured utilizing Debye–scherrer equation: D = kλ/β Cosθ. D is the average crystallite size (nm), k is the scherrer constant with value from 0.9 to 1, λ is the X-ray wavelength, βis the full width of half maximum and θ is the Bragg diffraction angle (degrees).

### Control of *F. oxysporum* by ZnO-NPs

#### Pathogen, inoculum and culture conditions

*Fusarium oxysporum* RCMB (008,002) was purchased from Regional Center for Mycology and Biotechnology (RCMB), Al-Azhar University, Cairo, Egypt. *F. oxysporum* was cultured on Czapek Dox agar (CDA) plates then incubated for 3–5 days at 25 ± 2 °C; Koch’s postulate was demonstrated for the pathogen confirmed as the causal agent of *Fusarium* wilt of eggplant **(**Al-Askar and Rashad 2010**)** and then kept at 4 °C for further use.

#### In-vitro assessment of antifungal activity and growth inhibition

##### Well diffusion method

Antifungal activity of biosynthesized ZnO-NPs was carried out using well diffusion method used by **(**Mohamed et al. 2021**)** with minor modifications. *F. oxysporum* was inoculated on PD broth medium, and then incubated at 28 ± 2 °C for 3–5 days. Fungal inoculum of *F. oxysporum* was spread on the surface of CDA plates. Then, eight wells with 8 mm diameter were made using sterile cork-borer on each agar plate (90 mm). The wells were filled with 100 µl of different concentrations of ZnO-NPs (7.81–1000 µg/ml) individually with triplicates. The culture plates were incubated at 25 °C for 7 days and the zones of inhibition were observed and measured.

##### Radial growth method

Radial growth of *F. oxysporum* was evaluated at different concentrations of ZnO-NPs (7.81, 15.62, 31.25, 62.5, 125, 250, 500 and 1000 µg/ml) according to method used by Joshi et al. (2019) with minor changes. Inhibition percentage of pathogen growth was calculated using the following equation:

$$\rm {Inhibition of pathogen growth }(\rm {\%})=\frac{\rm {Growth in the control}-\rm {Growth in the treatment}}{\rm {Growth in the control}}\times 100$$

#### In-vivo assessment efficacy of ZnO-NPs on S. melongena

The inoculum prepared according to Ortiz and Hoyos-Carvajal (2016) with minor modification as following, Sterilized 500 ml Erlenmeyer flask contains sterilized 250 ml PDA medium was inoculated with 3 discs 5.5 ml diameter with young mycelium (5 days), and then incubation for 7 days at 25 °C with stirring in shaker at 125 rpm under absence of light. On the other hand, pot experiment was carried out in the garden of Plant and Microbiology department, Faculty of Science, Al-Azhar University, Cairo, Egypt. *S. melongena* Balady seedling 15 day old were obtained from Legume Research Department, Field Crop Institute, Agricultural Research Center, Egypt. The sandy loam soil was autoclaved (1.5 atmosphere pressure, 121 °C for 30 min), and was distributed equally in disinfected pottery pots (30 cm in diameter), as one seedling per pot. The pots were arranged in a completely randomized design with six replicates as follows: treatment 1 (T1) the seedling sowing in sterilized soil, treatment 2 (T2) the seedling sowing in inoculated soil with *F. oxysporum*, treatment 3 (T3) the seedling sowing in sterilized soil and then seedling was spraying with 15 ml of ZnO-NPs (500 µg/ml) after 5, 20 days, treatment 4(T4) the seedling sowing in inoculated soil with *F. oxysporum* then seedling was spraying with 15 ml of ZnO-NPs after 5, 20 days. Disease development was recorded 15 days after sowing and disease severity was recorded. The plant samples were collected for biochemical indicators for resistance analysis when the plants were 45 days after sowing.

#### Disease symptoms and disease index

Disease symptoms were assessed 45 days after sowing and the disease index was evaluated according to Wang et al. (2016) using score consisting of five classes: 0 (no symptoms), 1 (slight yellow of lower leaves), 2(moderate yellow plant), 3 (wilted plant with browning of vascular bands), and 4 (plants severely stunted and destroyed) and coded as 0, 1, 2, 3, and 4 respectively. Percent of Disease index (PDI) was calculated according to the following formula: PDI = (1n_1_ + 2n_2_ + 3n_3_ + 4n_4_) X 100/4n_t_ Where; n_1_-n_4_ are the number of plants in the indicated classes and N_t_ is the total number of tested plants. Percent protection was calculated using following formula: Protection % = A–B/A × 100% Where, A = PDI in infected control plants B = PDI in infected- treated plants. Also shoot length, root length, fresh and dry weight.

#### Photosynthetic pigment determination

Pigments determined according to Vernon and Seely (1966), (1-g of fresh leaves was extracted by 100 ml of 80% aqueous acetone (v/v) then filtrated, then completed the volume to 100 ml using 80% acetone. The optical density of the plant extract was measured using spectrophotometer of three wavelengths (470, 649 and 665 nm). Pigments were calculated using the equations mentioned Mg chlorophyll (a)/g tissue = 11.63(A665)–2.39(A649), Mg chlorophyll (b)/g tissue = 20.11(A649)–5.18(A665), Mg chlorophyll (a + b)/g tissue = 6.45 (A665) + 17.72(A649).and Carotenoids = 1000 × O.D_470_−1.82 C_a_–85.02 C_b_/198 = mg/g fresh weight. “A” denotes the reading of optical density).

#### Determination of the content of osmolytes

Total soluble carbohydrate was extracted as the following: 1 g of the dried plant shoot to be analysed was put in 100 ml capacity conical flask, to which 5 ml of 2% phenol water and 10 ml 30% trichloroacetic acid were added. The mixture was shaken and kept overnight before being filtered; the filtrate was made up to 50 ml then The soluble sugar content was calculated by the method described by Irigoyen et al. (1992). Total soluble protein of plants (1 g of the dried leaves add to 5 ml of 2% phenol water and 10 ml distilled water was added, shaken and kept overnight, filtered and complete volume to 50 ml with distilled water, then The soluble protein content of the dry shoot was determined according to Lowry et al. (1951).

#### Determination of total phenol content

One gram of dry leaves was extracted with 80% cold methanol (v/v) for three times at 0 °C. The extract was filtered; the volume of sample was completed to 25 ml with cold methanol. The development color was reading at 725 nm. Using 0.5 ml 80% ethanol and reagents only as a blank, then Total phenol content was determined according to **(**Dai et al. 1993; Hashem et al. 2021a, b**)**.

#### Assay of antioxidant enzymes activity

Peroxidase activity (POD) was assayed according to that method described by Bergmeyer and Bernt (1974) with modifications by Badawy et al. (2021). Activity of polyphenol oxidase enzyme was calculated by the procedure used by Matta and Dimond (1963).

#### Isozymes electrophoresis

Native polyacrylamide gel electrophoresis (Native-PAGE) isozyme electrophoresis was performed in leaf (100 mg fresh weight) samples were estimated and evaluate the peroxidase (POD) isozyme **(**Bradford 1976**)**. The isozyme polyphenol oxidase (PPO) was calculated according to the Barceló et al. (1987) approach. The run was performed at 150 V until the bromophenol blu dye has reached the separating gel and then the voltage was increased to 200 V. The electrophoretic apparatus was placed inside a refrigerator during the running duration then Gel was placed into a solution composed of Benzidine di HCl 0.125 gm, Glacial acetic acid 2 ml, Distilled water up to 50 ml and 5 drops of hydrogen peroxide were added. The gel was incubated at room temperature until bands appeared for POD **(**Brown 1978**)**. After electrophoresis, the gel was soaked in 0.1 M Sodium Mono Di Phosphate buffer (pH 6.8) solved in 100 mg Sulfanilic acid, then mixed with 30 mg Cathecol solved in 1 ml aceton. The gel incubated at 37 °C until bands appear for PPO determination.

### Statistical analysis

One-way variance analysis (ANOVA) was applied to the resulting data. Least significant difference (LSD test) using CoStat (CoHort, Monterey, CA, USA) was used to demonstrate statistically relevant differences between treatments at p < 0.05. Results shown as mean ± standard errors (n = 3) (Snedecor and Cochran 1980).

## Results and discussion

### Synthesis and characterization of ZnO-NPs

The green biosynthesis of ZnO-NPs has been gained an importance suggested as possible alternatives to chemical and physical methods. Metabolites secreted by *P. expansum* have effective in the formation of ZnO-NPs, besides stabilizing this formed NPs. *P. expansum* was utilized as a bio-reactor for formation of ZnO-NPs through harnessing bioactive macromolecules secreted therefrom *P. expansum* (Fouda et al. 2018). Appearance of milky white color after contacting of filtrate of *P. expansum* with precursor (zinc acetate) at the reaction complete indicated ZO-NPs formation. After calcination ZnO-NPs were obtained as the white powder at 85 °C for 48 h.

**Fig. 1:**
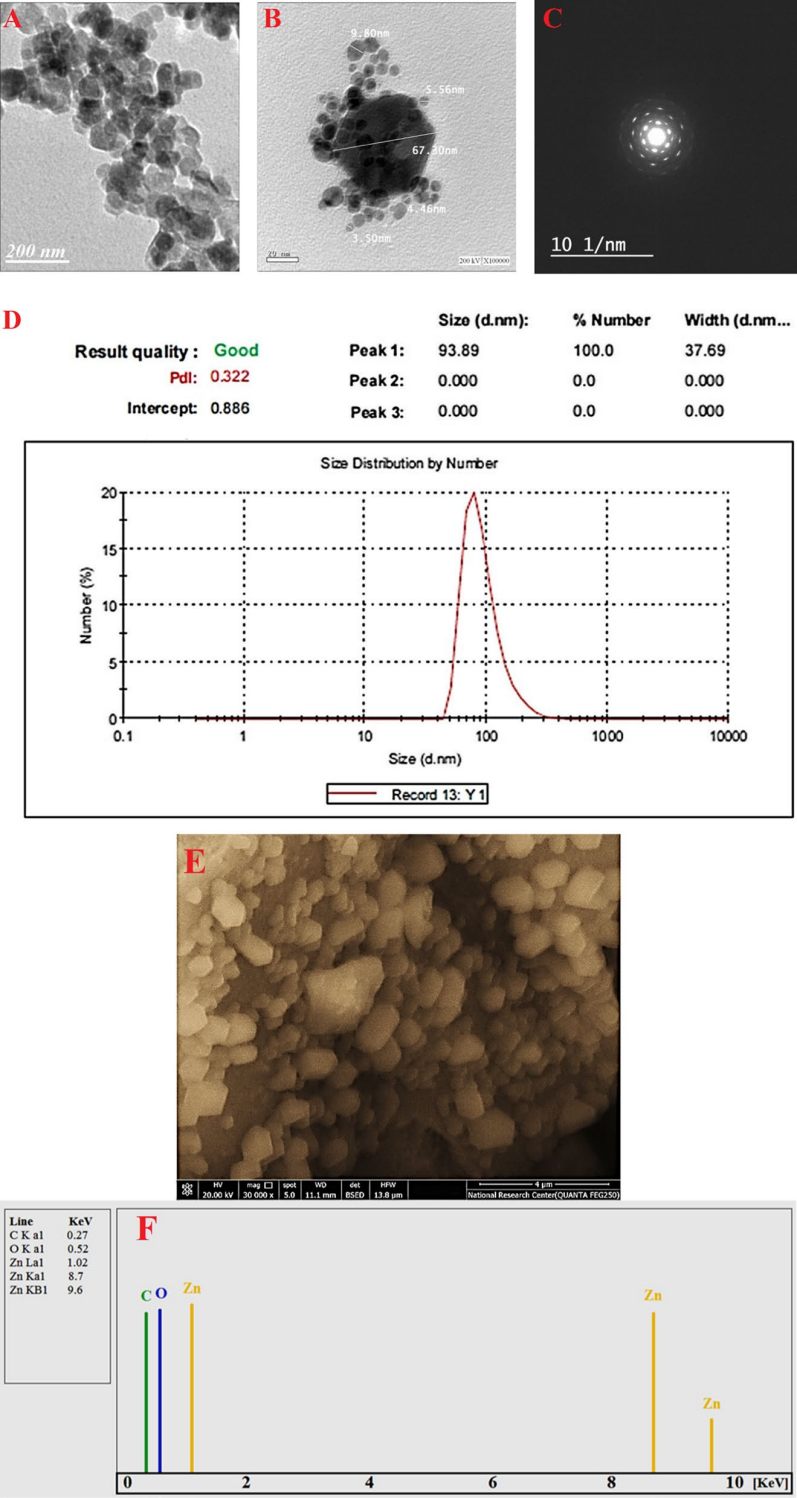
Characterization of ZnO-NPs synthesized by *P. expansum*; **A** and **B** TEM micrograph, **c** SAED pattern, **D** DLS, and **E** and **F** SEM–EDX spectra of biosynthesized ZnO-NPs

The characterizations of biosynthesized ZnO-NPs were proceed using TEM, DLS, and SEM–EDX analysis to investigate the shape, size, particle size distribution, and quantitative elemental analysis of ZnO-NPs. Figure 1A and B showed successful fabrication of hexagonal ZnO-NPs through harnessing metabolites involved in filtrate of *P. expansum*, with average size ranging between 3.50 and 67.30 nm. Figure 1C showed the area selected electron-diffraction (SAED) patterns of the ZnO-NPs, indicating good sharp-rings, which reveal the hexagonal crystalline nature of the ZnO-NPs. Figure 1D represented the average particle size-distributions in nano-sol acquired from DLS for ZnO-NPs synthesized *by P. expansum*. The obtaining data showed that, the average size of 100% ZnO-NPs was found to be around 94 nm for synthesized by *P. expansum* with high polydispersity. The sizes measured by DLS not depend only on metallic core of ZnO-NPs but also affected with substances absorbed on ZnO-NPs surfaces as stabilizers agent. According to (Mohamed et al. 2019) reported the successful formed hexagonal ZnO-NPs with size range 10–42 nm using TEM, while the distribution of the ZnO-NPs in nano-sol was 163.34 nm using DLS; it was biologically formed using *F. keratoplasticum* filtrate. Other reports showed a difference in shape and size of ZnO-NPs biologically formed by *A. terreus*, *A. niger,* and *P. chrysogenum* (Fouda et al. 2018; Mohamed et al. 2019, 2021). On the other hand, the qualitative and quantitative analysis of biosynthesized ZnO-NPs were achieved using SEM–EDX to detect elemental compositions of ZnO-NPs with relative percentages such as weigh and atomic % Fig. 1E and F. SEM clearly shows distributed ZnO-NPs with agglomeration of NPs. High peaks in EDX results reflect the highest concentration of the element to be identified or studied. Obviously, an EDX spectrum contains mainly Zn and O element with weight percentages 64.54 and 19.75% respectively. The presence of C may be due to the fungal biomolecules involved in biomass filtrate. In the same line, (Mohamed et al. 2021) reported that the main peaks of EDX spectra for ZnO-NPs synthesized by *P. chrysogenum* were Zn (58.3%) and O (20%), in addition the presence of other peaks related to biomolecules in *P. chrysogenum* filtrate which conjugated with ZnO-NPs.

**Fig. 2:**
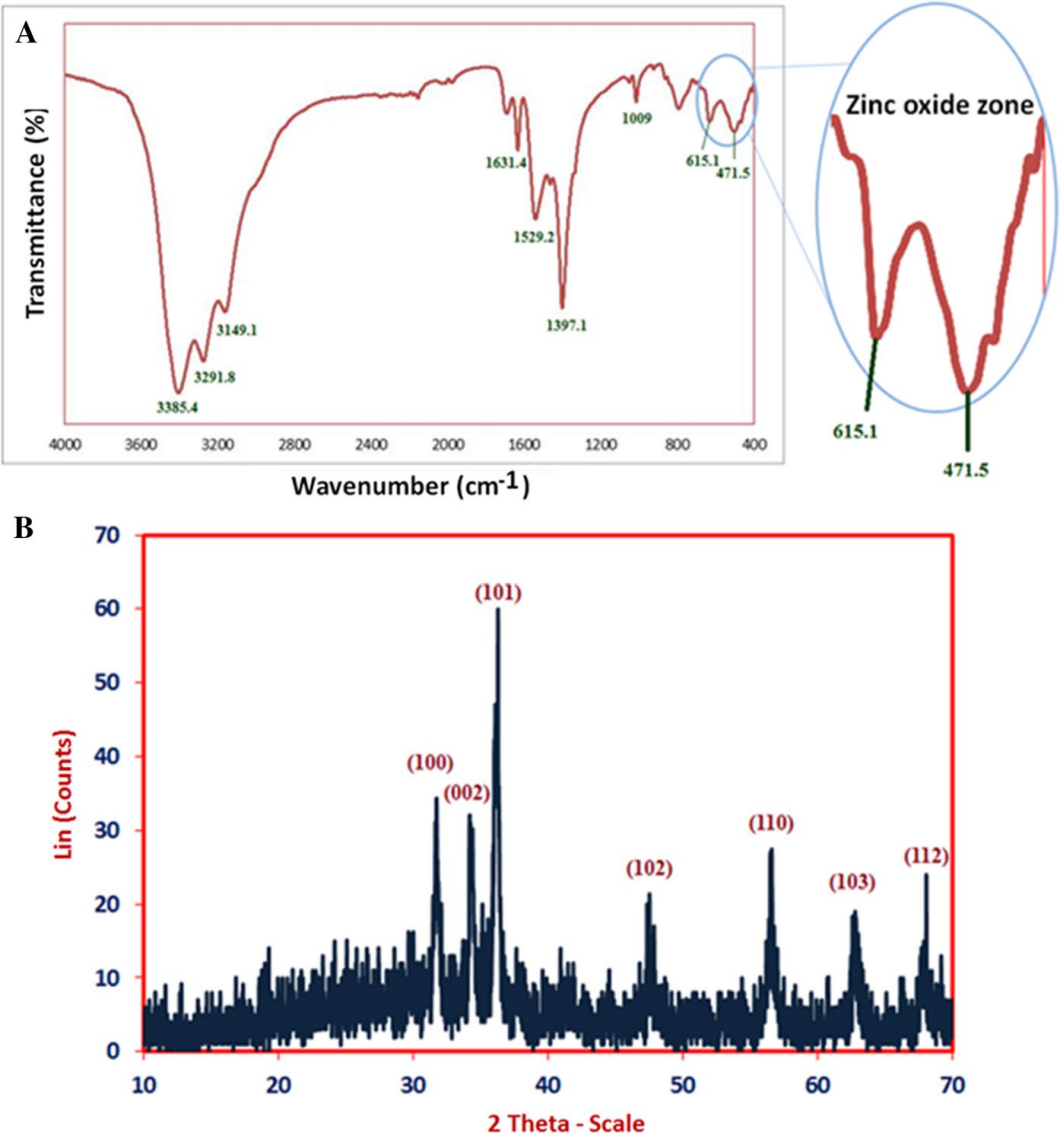
**A** FTIR spectra and **B** XRD pattern of biosynthesized ZnO-NPs

The functional groups those found in ZnO-NPs are characterized using FT-IR analysis. As seen in Fig. 2A. The interaction of a capping agent from *P. expansum* extract with ZnO-NPs is indicated by wave numbers at 3385.4, 3291.8, 3149.1, 1631.4, 1529.2, 1397.1, 1009, 615.1 and 471.5 cm^−1^. FT-IR of biogenic ZnO-NPs showed different peaks at 3385.4, 3291.8, and 3149.1 cm^−1^ (O–H, N–H stretching, aliphatic primary amines and O–H broad stretching) (Mohamed et al. 2021). Other peaks are appeared at 1631.4 cm^−1^ and 1529.2 cm^−1^ (amide I band), 1397.1 and 1009 cm^−1^ (C-N stretching of amines) (Mohamed et al. 2019). Interestingly, the FT-IR peaks at 615.1 and 471.5 cm^−1^ correspond to formation zone of Zn–O (Fouda et al. 2018; Mohamed et al. 2021). X-ray diffraction pattern are crucial tool for confirms the crystalline nature of green synthesized ZnO-NPs. As seen in Fig. 2B, XRD based ZnO-NPs characterization exhibit nine peaks at 2 θ values 31.7°, 34.2°, 36.1°, 47.4°, 56.5°, 62.5°, and 68° which assigned to planes 100, 002, 101, 102, 202, 110, 103, and 112 respectively for ZnO-NPs. The visualized XRD peaks are matched with JCPDS number: 01-089-0510 of crystallo-graphic ZnO-NPs. In line with our clarification of the results, (Fouda et al. 2020) reported that the successful fabrication of crystallite, monoclinic phase ZnO-NPs at the same XRD diffraction planes utilizing metabolites of fungal. The average sizes of crystallite ZnO- particles were calculated using scherrer’s equation. In this context, the size of ZnO- particles ranged between 14.2 and 107.5 nm, output from the analysis of the equation.

### In vitro control of *F. oxysporum*

#### Antifungal activity and minimum inhibitory concentration

**Fig. 3:**
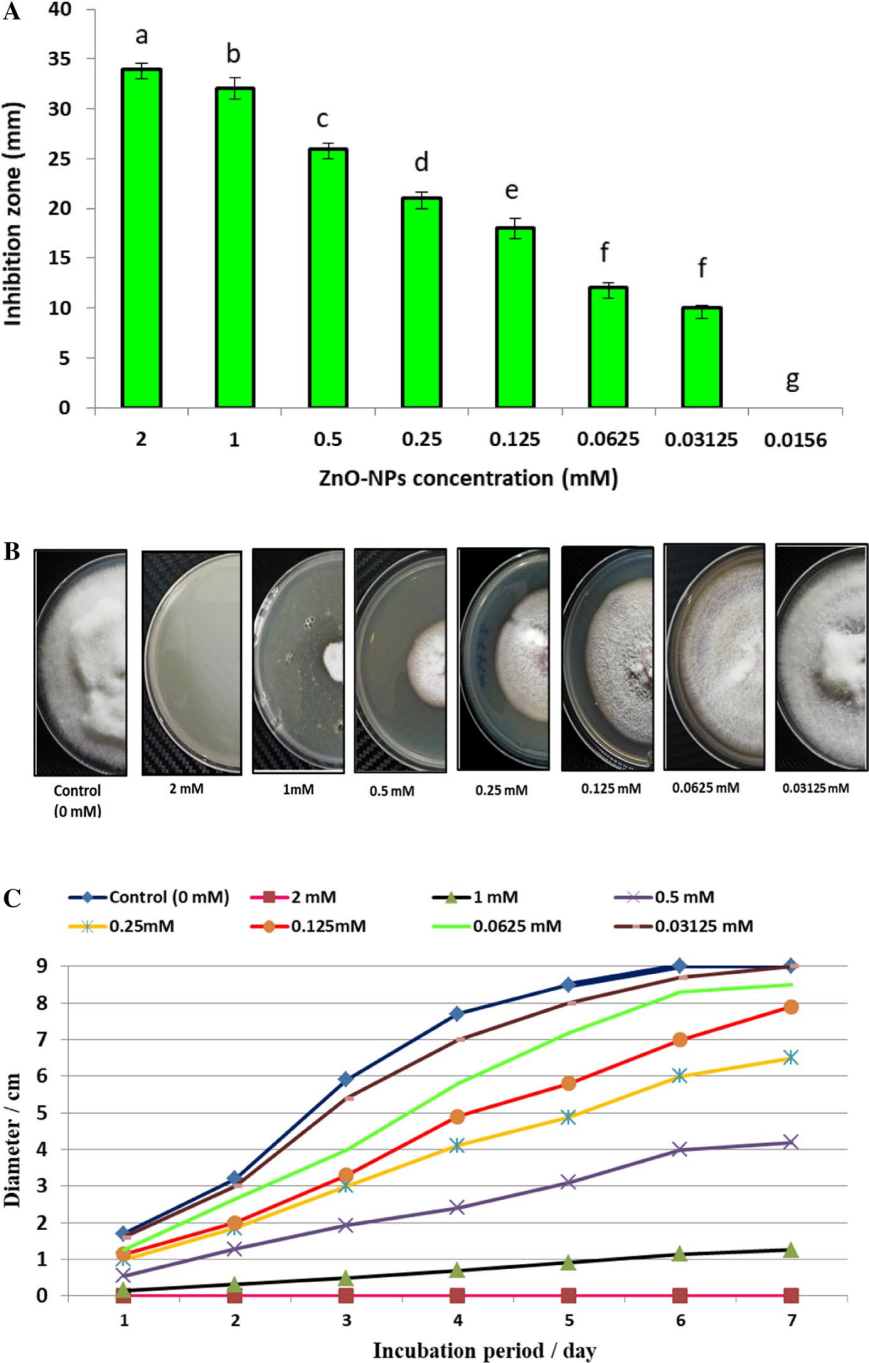

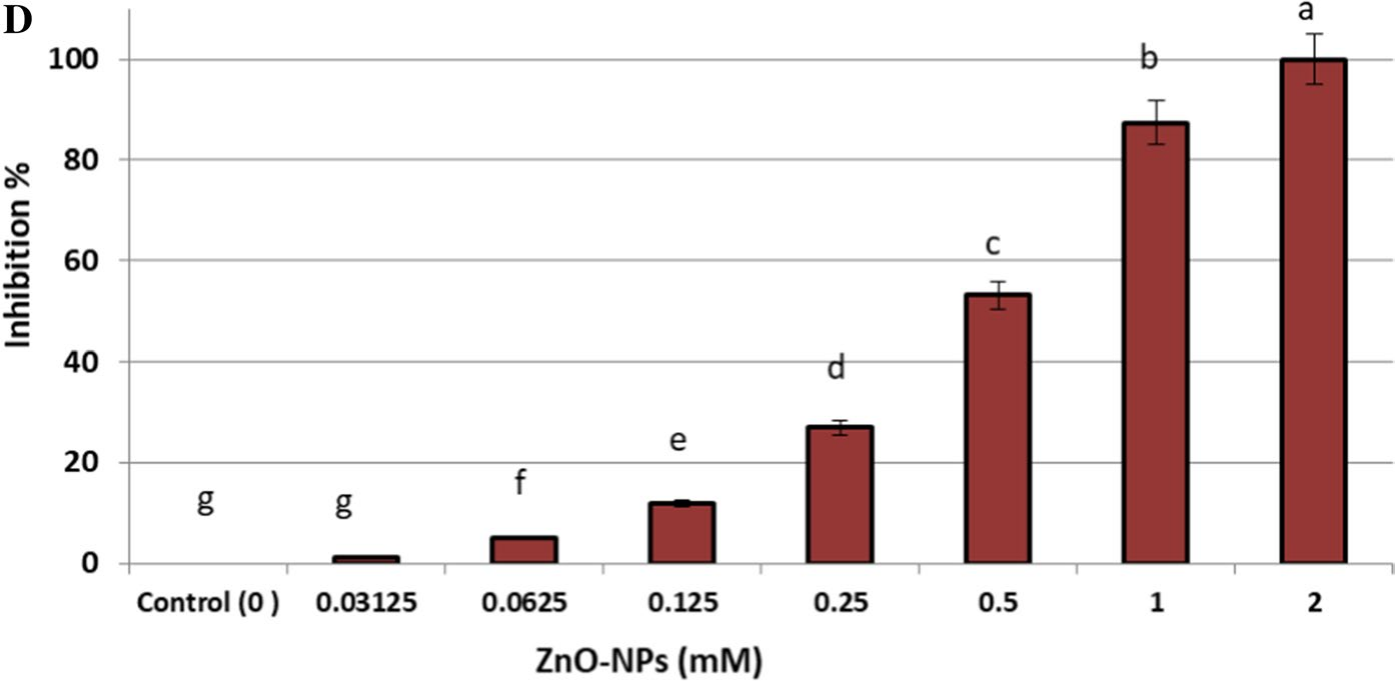
In *vitro* control of *F. oxysporum* using biosynthesized ZnO-NPs (**A–D**): **A** Antifungal activity and MIC; **B** Linear growth on PDA agar plates at 7th day; **C** Linear growth at different incubation period (1–7 days); **D** Growth inhibition at 7th day

Recently, biosynthesized metal nanoparticles are wildly used in control of fungal plant pathogens (Dimkpa et al. 2013; Elmer et al. 2018; Kalia et al. 2020). In the current study, biosynthesized ZnO-NPs were evaluated as antifungal toward *F. oxysporum* as shown in Fig. 3A. Results revealed that, ZnO-NPs (1000 µg/ml) exhibited a promising antifungal activity against *F. oxysporum* in vitro, where inhibition zone was 34.4 ± 0.58 mm. Moreover, concentrations 500, 250, 125, 62.5, 31.25 and 15.62 µg/ml exhibited inhibition zones but lower than 1000 µg/ml where were 30.6 ± 1.15, 25.3 ± 0.57, 20.6 ± 0.57, 17.0 ± 1.0, 11.5 ± 0.5 and 9.8 ± 0.29 mm respectively. Hence, MIC of ZnO-NPs against *F. oxysporum* was 15.62 µg/ml.

#### Linear growth and minimum fungicidal concentration

Effect of different concentrations of ZnO-NPs on linear growth of *F. oxysporum* at different incubation periods (1–7 days) was evaluated as shown in Fig. 3B and C. Linear growth of *F. oxysporum* was performed to determine the inhibition percentage for each concentration of ZnO-NPs. Results illustrated that, *F. oxysporum* could not grow on the surface of PDA agar plate which amended by 1000 µg/ml of ZnO-NPs, where inhibition percentage was 100% as shown in Fig. 3D. Moreover, the growth diameter is increased with decreasing the concentration of ZnO-NPs, but inhibition percentage is decreased. Inhibition percentages of ZnO-NPs at concentrations 500, 250, 125, 62.5, 31.25 and 15.62 µg/ml were 87.37 ± 1.48, 53.11 ± 1.01, 26.9 ± 0.88, 11.9 ± 0.37, 5.16 ± 0.29 and 1.33 ± 0.29% respectively. On the other hand, the concentration 7.81 µg/ml did not give any inhibition for *F. oxysporum.* From these data, the concentration 1000 µg/ml was the minimum fungicidal toward *F. oxysporum*. Yehia and Ahmed (2013) reported growth inhibition of ZnO-NPs at concentration 1200 µg/l against *F. oxysporum* was 77%. According to (González-Merino et al. et al. 2021) ZnO-NPs have antifungal action, suppressing mycelial development and sporulation of *F. oxysporum* in *vitro*. Eventually, biosynthesized ZnO-NPs was effective toward *F. oxysprum* in vitro, where MIC and MFC were 15.62 and 1000 µg/ml respectively.

### In vivo control of *F. oxysporum*

#### Efficacy of ZnO-NPs on F. oxysporum wilt disease of S. melongena under pot Conditions

**Fig. 4:**
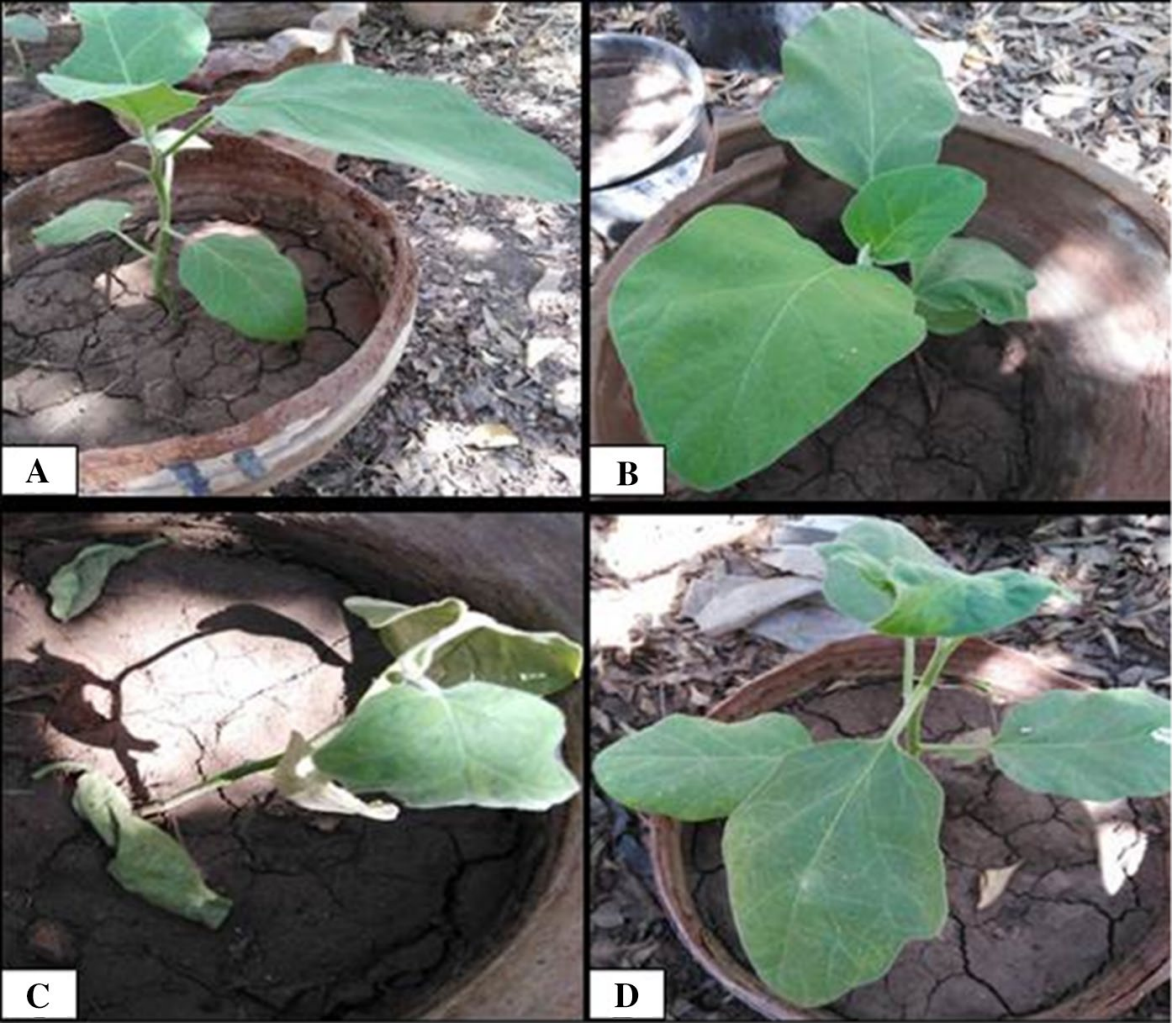
Efficacy of ZnO-NPs on *F. oxysporum* wilt of *S. melongena* under pot Conditions: **A** control healthy, **B** healthy treated with ZnO-NPs, **C** control infected and **D** infected treated with ZnO-NPs

Results presented in Table 1 indicated that *F. oxysporum* caused The emergence of severe symptoms on the *S. melongena* plant 83.33% compared with healthy control which no wilt symptoms appeared Fig. 4. These results confirmed that Eggplant melongena ( *S. melongena*) Balady is susceptible to *F. oxysporum*. These results explained by (Abada et al. 2018) they reported that The fungus *F. oxysporum* is the most destructive causal pathogen causing *S. melongena* wilt by invades the vascular vesicles and blocking the xylem transport vesicle (s) and causes severe wilting. Application of the ZnO-NPs by folair spraying to *S. melongena* infested with fungus *F. oxysporum* showed greater potency in controlling the pathogen that decrease diseas index to 20.83 and increase protection of *S. melongena* against *F. oxysporum* wilt disease. These results confirmed by (Yehia and Ahmed 2013) they reported that the antifungal activity of ZnO-NPs against *Fusarium oxysporum* and mycotoxin fusaric acid.

**Table 1:** Effect of ZnO-NPs on *F. oxysporum* wilt disease of *S. melongena* under pots conditions

Treatment	Disease symptoms classes					DI (disease index) (%)	Protection (%)
	0	1	2	3	4		
Control healthy	6	0	0	0	0	0	–
Control Infected	0	0	1	2	3	83.33	0
Healthy treated with Nano	6	0	0	0	0	0	–
Infected treated with Nano	2	3	1	0	0	20.83	75

#### Morphological indicators responses of *S. melongena* to ZnO-NPs under pot conditions

Results presented in Table 2 indicated that all investigated growth parameters (shoot and root length, fresh and dry weight plant biomass), of infected *S. melongena* plants with *F. oxysporum* were significantly decreased compared with healthy control plants.these results similar to (Abada et al. 2018) they reported that *F. oxysporum* were significantly decreased *S. melongena* plant height as well as fresh and dry plant biomass. Results in Table 2 showed application of ZnO-NPs resulted to increasing of root as shoot length and fresh and dry weight. These results explained as Zinc one of the essential micronutrients required for growth of plant, and the only metal exists in all six enzyme classes: transferases, ligases, hydrolases, oxidoreductases, lyases and isomerases (Auld 2001). These results also indicated by (Thunugunta et al. 2018) they reported that ZnO NPs enhanced *S. melongena* growth under greenhouse conditions. Application of ZnO NPs significant increasing tested growth parameters compared with infected control similar to (Yehia and Ahmed 2013) The antifungal potency of ZnO-NPs against *F. oxysporum* and prevented fusaric acid mycotoxin synthesis by deformation the growing mycelia of *F. oxysporum*. Also (Singh et al. 2018) proved antimicrobial activity of ZnO-NPs are considered as a bio-safe material for stimulation of seed germination and plant growth as well as disease suppression and plant protection. There are another mechanisms as ZnO-NPs caused reduction of mycelial growth of *F. oxysporum*, antifungal activity may be due to suppression of metabolites that required for pathogenesis (Dimkpa et al. 2013).

**Table 2:** Effect of biogenic ZnO-NPs on morphological indicators of *S. melongena* under pots conditions

Treatments	Plant height (cm)	Root length (cm)	Shoot F. wt. (g)	Shoot D. wt. (g)	Root F. wt. (g)	Root D.Wt. (g)
Control (H.)	34 ± 2.56^ab^	11 ± 1.73^a^	9.76 ± 1.36^a^	2.01 ± 0.75^a^	2.43 ± 0.51^a^	0.51 ± 0.09^a^
Control (Inf.)	13.33 ± 1.52^c^	5 ± 1^b^	2.26 ± 0.8^c^	0.25 ± 0.07^b^	0.5 ± 0.2^b^	0.04 ± 0.005^b^
Healthy treated with Nano	37.67 ± 2.52^a^	12 ± 1.73^a^	10.63 ± 1.58^a^	2.34 ± 1.2^a^	3.03 ± 0.31^a^	5.13 ± 0.2^a^
Infected treated with Nano	33.66 ± 1.52^b^	10.33 ± 2.08^a^	5.56 ± 1.69^b^	1.28 ± 0.46^ab^	0.93 ± 0.51^b^	0.24 ± 0.1^b^
L. S. D. at 0.05	3.994	3.169	2.643	1.458	0.809	0.2419

Values are means ± SD (n = 3). Data within the groups are analyzed using one-way analysis of variance (ANOVA) followed by

^a,b,c,d^ Duncan’s multiple range test (DMRT), *LSD* least significant differences

#### Effect of ZnO-NPs on photosynthetic pigments of *S. melongena* under pots conditions

**Fig. 5:**
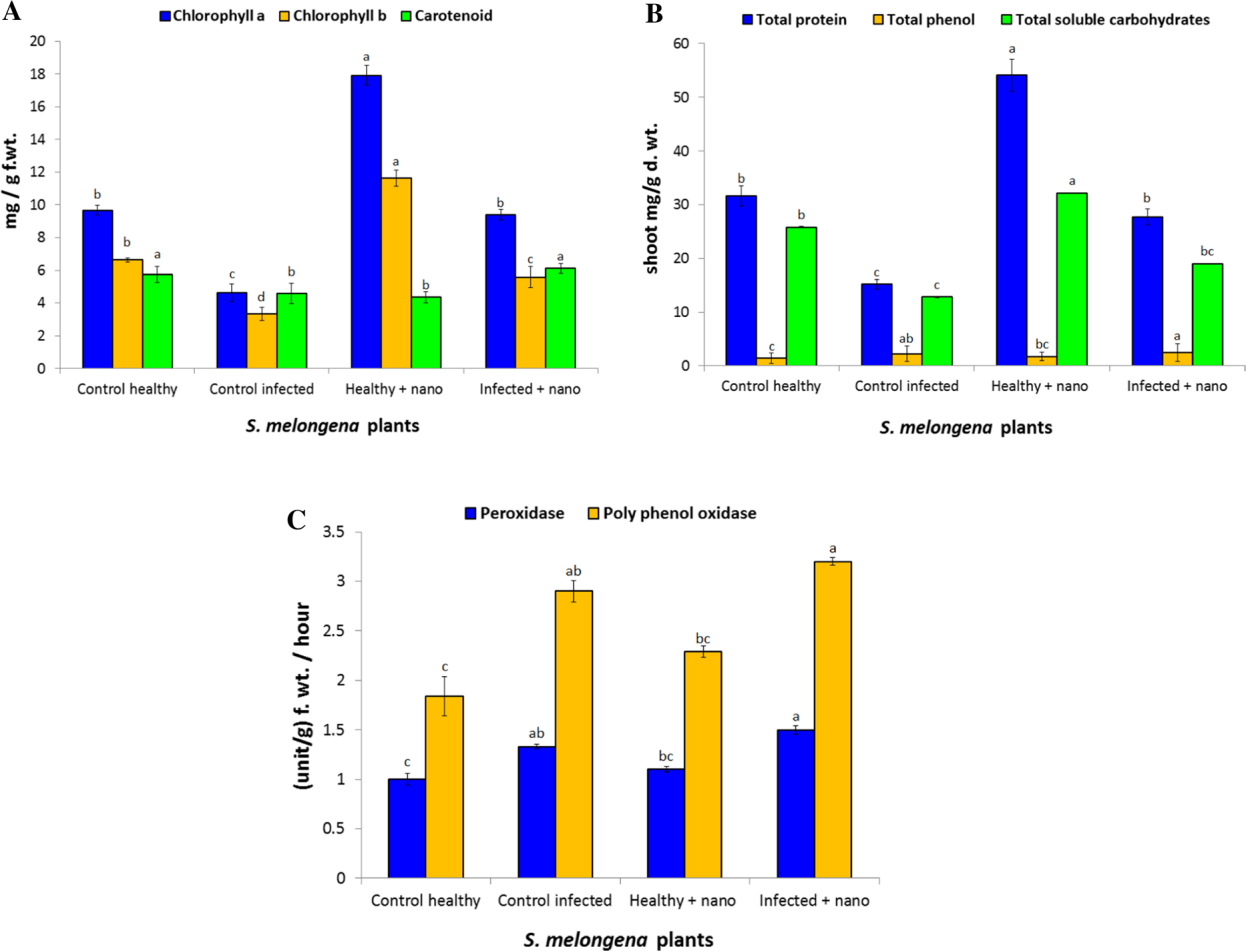
Effect of ZnO-NPs on the metabolic indicators of (*S. melongena)*: **A** photosynthetic pigments, **B** phenol, protein and soluble carbohydrate and **C** antioxidant enzymes

The observed results in Fig. 5A clearly revealed that, chlorophyll a and b as well as carotenoids contents were highly significantly decreased in infected *S. melongena* plants with *F. oxysporum* by 52.09% and 49.84% respectively compared to healthy control. The decrease in chlorophyll content may be due to the generation of reactive oxygen species (ROS) causing damage to chlorophyll a and the plant photosynthesis will inhibited (Aldinary et al. 2021). Results in Fig. 5 indicated application of ZnO-NPs resulted to significant increasing of photosynthetic pigments in healthy and infected *S. melongena* plant. Our results in line with many resent studies (Latef et al. 2017; Bala et al. 2019) they recorded that ZnO-NPs resulted in enhanced photosynthetic pigments contents.

#### Effect of ZnO NPs on the metabolic indicators of (*S. melongena*)

Data Fig. 5B showed that, *Fusarium* infection caused significant increase in total phenols. It was noticed that, *S. melongena* plants treated with ZnO-NPs showed significant increase in total phenols of both healthy and infected plants. These results similar to many studies (Ghorbanpour and Hadian 2015; Večeřová et al. 2016) they recorded increasing phenolic content in treated plant with NPs. This increasing in phenolic contents explained antifungal activity against *F.oxysporum* through several mechanisms as (i) cell rupture; (ii) release of intracellular proteins and carbohydrates that inhibit fungal growth; (iii) reduction of ATP production and (iv) oxidative lesions; and chelation of iron ions (Boyer et al. 1988; Maringgal et al. 2019). The amount of total phenolics was significantly increased upon exposure to ZnO-NPs Due to the ZnO-NPs have ability to generate ROS, thus phenolics acts as important antioxidants and protective compounds (Balážová et al. 2020).

Infected *S. melongena* plants with *F. oxysporum* showed significant decreases in contents of soluble carbohydrate. Application of ZnO-NPs resulted in significant increasing of soluble carbohydrate in both healthy and infected *S. melongena* compared to control (Fig. 5B). These Soluble carbohydrate are involved in the responses to stresses, and act as metabolic signaling that activate specific or hormonal- crosstalk transduction pathways, resulting in gene expression modifications (Couée et al. 2006).

Results in Fig. 5B showed that total soluble proteins in shoot were significantly decreased in *S. melongena* plants due to the infection with *F.oxysporum*. these results agree with (Boccardo et al. et al. 2019) they recorded that pathogen attack resulted in a reduction of shoot soluble protein in infected plants. These inhibition in total soluble protein explained by inhibition the process of protein synthesis or the pathogens consume nitrogen which could have been utilized for synthesizing proteins (Siddique et al. 2014). For more application of ZnO-NPs resulted to increase of total soluble protein compared with untreated plants. These similar to (Brunner et al. 2006) they reported that the protein content of leaves treated with ZnO-NPs showed a significant increase in comparison with control. The activation of the host defense mechanisms as an indicator of resistance resulted from Increasing protein content (Siddique et al. 2014).

#### Effect of ZnO NPs on oxidative enzymes of *S. melongena* under pots conditions

Results in Fig. 5C showed increasing of peroxidase (POD) and polyphenol oxidase (PPO) in infected *S. melongena* plant with *F. oxysporum* than healthy. These results similar (Altinok and Dikilitas 2014) to they reported infection of *S. melongena* with *F. oxysporum* showed significant increases in the activity of antioxidant enzymes as polyphenol oxidase. This increasing of antioxidant enzymes to keep reactive oxygen species (ROS) at the lower level in the cell and keeps cells from destroying (Gill and Tuteja 2010). For more application of ZnO-NPs resulted to significant increasing of antioxidant enzymes POD as well as PPO in both healthy and infected. Similar to (Latef et al. 2017; Abdelaziz et al. 2021) they reported that Treatment plants with ZnO-NPs increased the activity of antioxidant enzymes.

#### Effect of ZnO-NPs on antioxidant isozymes (peroxidase and polyphenol oxidase) of *S. melongena* under pots conditions

**Fig. 6:**
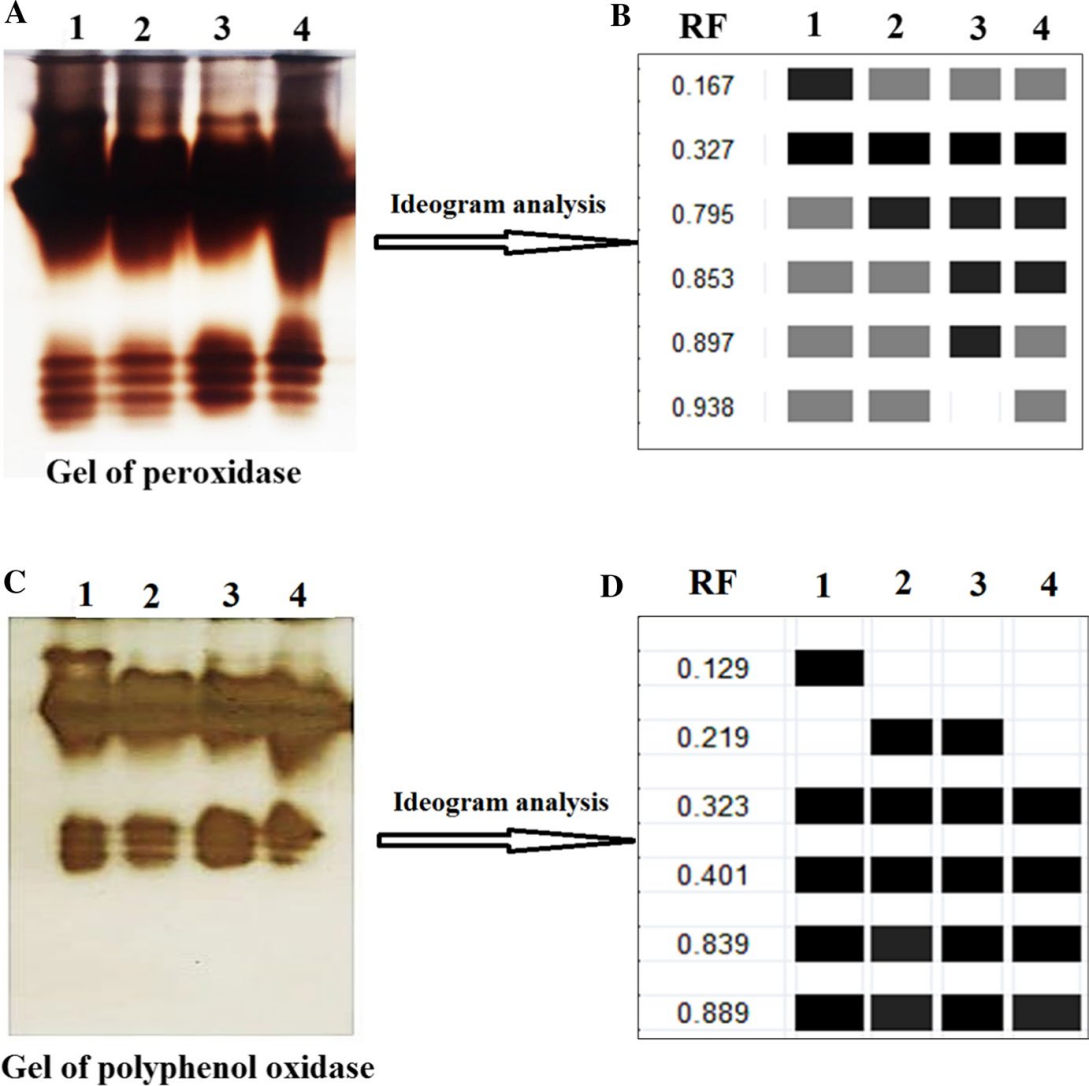
Effect of ZnO-NPs on healthy and infected *S. melongena*: 1- control healthy, 2- control infected, healthy treated with ZnO-NPs and 4- infected with ZnO-NPs where, **A** Gel of peroxidase isozyme image, **B** Ideogram analysis of peroxidase isozyme, **C** Gel of polyphenol oxidase isozyme image and **D**: deogram analysis of polyphenol oxidase isozyme

Results in Fig. 6 indicated that *S. melongena* showed variation in relative mobility and density polypeptide bands of Peroxidase by 28.571% Polymorphism as pathogenicity indicators and or treatment with ZnO-NPs, where healthy control as well as infected and healthy with ZnO-NPs gave 6 bands of POD isozymes POX1: POD6(0.176, 0.327, 0.795, 0.853, 0.897, 0.938 and 0.942), but ZnO-NPs infected plant gave 5. Also results in Fig. 6 showed that healthy plant treated with ZnO NPs had unique band. For more, *S. melongena* showed variation in relative mobility and density polypeptide bands of Polyphenol Oxidase by 33.33% Polymorphism as pathogenicity indicators and or treatment with ZnO NPs, where healthy control as well as infected and healthy with ZnO-NPs gave 5bands of PPO isozymes PPO1: PPO5 (0.129, 0.219, 0.323, 0.401, 0.839 and 0.889), but ZnO-NPs infected plant gave 4. Also results in Fig. 6 showed unique band in healthy control at 0.129 relative mobility. These results similar to Abdelaziz et al. et al. (2021) they recoded that infection with *F. oxysporum* resulted to increasing isozymes but ZnO-NPs inhibited isozymes. Nano particles act as promoter and/or stressor enhanced the antioxidant defense systems of plants which resulted to the improvement of plant tolerance through scavenging system of excess reactive oxygen species (ROS) (Hussein et al. 2019).

## Conclusion

In the current study, fast green eco-friendly method was used to synthesize ZnO-NPs by *Penicillium expansum* ATCC 7861. Based on the results presented here, it can be concluded that ZnO-NPs mycosynthesized can be used as a promising and safe alternative antifungal agent against *F. oxysporum* in vitro and in vivo. However, application of biosynthesis ZnO-NPs significantly provides the potential to overcome *Fusarium* wilt disease in eggplants through inhibiting the phytopathogenic fungus, enhancement of ROS detoxification by more effective antioxidant defense systems, improvement of morphological, molecular, and enzymatic aspects.
